# Psychometric properties of the Usability and Acceptability Scale (UAS) for evaluating digital tools in children and adolescent users

**DOI:** 10.3389/fpsyg.2026.1702085

**Published:** 2026-04-07

**Authors:** Matilde Spinoso, Mariagrazia Benassi, Noemi Mazzoni, Matteo Orsoni, Luca Stefanutti, Pasquale Anselmi, Debora de Chiusole, Alice Bacherini, Irene Pierluigi, Sara Garofalo, Giulia Balboni, Sara Giovagnoli

**Affiliations:** 1 Department of Psychology “Renzo Canestrari”, Cesena Campus University of Bologna, Cesena, Italy; 2 Department of Philosophy, Sociology, Education, and Applied Psychology, University of Padua, Padua, Italy; 3 Department of Philosophy, Social Sciences, and Education, University of Perugia, Perugia, Italy

**Keywords:** acceptability, children and adolescents, digital tools, psychometric properties, usability, usability questionnaire, users scale

## Abstract

The rapid integration of digital tools in educational and clinical settings highlights the need for assessing their usability and acceptability, particularly among populations of developmental age. This study aims to validate the Usability and Acceptability Scale (UAS), a new scale tailored for children aged 4 to 18, derived from integration and an adaptation of the Technology Acceptance Model Scale and System Usability Scale. The UAS was administered to a sample of 908 participants. Results of Exploratory and Confirmatory Factor Analyses consistently supported a bifactorial model encompassing the two dimensions of usability and acceptability. Reliability was also assessed using Cronbach’s *α* and McDonald’s *ω*, with overall results indicating acceptable internal consistency. These findings suggested that the UAS is a valid and reliable questionnaire for evaluating digital tools among younger users, offering valuable insights for developers and educators aiming to create child-friendly technologies.

## Introduction

Nowadays, technological advancements are progressing at an unprecedented rate compared to previous times. Technology plays an increasing role in children’s lives, shaping their learning, entertainment, and social interactions. In this context, the usability and acceptability of new technologies are particularly relevant for the effectiveness and implementation of new digital tools, such as computerized neuropsychological tests (Davis, 1989; Venkatesh and Davis, 2000). Concerning the latter, it is essential to ensure that technology not only enhances assessment quality but is also perceived as accessible and easily usable by children (Druin, 2002). Acceptability refers to the extent to which a user finds a computer system suitable, agreeable, and satisfactory. It is closely linked to the concept of usability, which measures the extent to which specified users can use a product to achieve specific goals with effectiveness, efficiency, and satisfaction in a defined context of use (ISO, 1998).

Although usability and acceptability are closely related constructs, they are conceptually distinct. Usability refers to the system’s functional capacity to support effective and efficient task performance (performance-oriented; ISO, 1998). In contrast, acceptability reflects users’ cognitive and motivational beliefs influencing their intention to engage with the system (motivation-oriented; Venkatesh and Bala, 2008). While both constructs involve the notion of “ease,” usability concerns the objective support provided during task execution, whereas perceived ease of use within the acceptance framework reflects the user’s subjective belief that interacting with the system requires little effort.

Assessing the acceptability and usability of digital technologies in children is crucial for several reasons. Children have distinct cognitive and motor skills compared to adults, which significantly influence their interaction with technology (Piaget, 1970; Diamond, 2000). Therefore, tools designed for children must be tailored to their specific needs and capabilities (Markopoulos et al., 2008; Hourcade, 2007). Additionally, children’s attitudes toward technology can affect their willingness to engage with digital tools, impacting the effectiveness of educational and therapeutic interventions (Druin, 2002). Moreover, understanding the usability and acceptability of these technologies can help developers create more engaging and effective products, ultimately enhancing the overall user experience for children. To date, to effectively assess usability and acceptability various methods are employed, including usability testing methods (e.g., the System Usability Scale (SUS), and task completion rates; Brooke, 1996; Rubin and Chisnell, 2008), direct observation techniques (e.g., think-aloud protocols; Nielsen, 1994; van den Haak et al., 2003), heuristic evaluation based on established design principles (e.g., Nielsen’s heuristics; Nielsen and Molich, 1990; Jeffries and Desurvire, 1992), cognitive walkthrough approaches (e.g., Polson et al., 1992; Wharton et al., 1994) and focus groups (e.g., contextual interviews and group discussions; Kuniavsky, 2003). Among these approaches, questionnaires represent one of the most widely used tools in the field (Brooke, 1996; Bangor et al., 2008). When applied to pediatric populations, these traditional methods require substantial adaptations to accommodate children’s developmental characteristics. Specifically, the assessment of usability and acceptability in children draws on four main categories of methods: observational, verbalization, survey, and longitudinal approaches (Markopoulos et al., 2008). Observational methods range from passive to participatory, with the moderator choosing whether or not to engage with the participant and may be complemented by additional tools such as eye-tracking to monitor interface interaction (Masood and Thigambaram, 2015). Verbalization methods capture what participants say, either spontaneously or upon request, and include concurrent and retrospective think-aloud protocols (Donker and Markopoulos, 2002), active and robotic intervention, and post-task interviews, which are particularly useful for collecting immediate data (Baauw and Markopoulous, 2004).

Collaborative techniques such as co-discovery and peer tutoring are employed to facilitate more natural communication between children (Downey, 2007; Markopoulos and Bekker, 2003; Ognjanovic and Ralls, 2013). For interactive technology products, the Wizard-of-Oz method allows a moderator to control interactivity remotely and is particularly useful in early development stages (Höysniemi et al., 2004). Longitudinal methods such as diary studies allow observation of product use in naturalistic contexts over time, although their implementation in applied settings may be demanding (Markopoulos et al., 2008; Jacoby, 2011).

Survey methods include questionnaires and interviews, which can be combined to clarify ambiguous responses (Markopoulos et al., 2008). Child-friendly adaptations, such as the Fun Toolkit, increase engagement and reduce satisficing (Read, 2008; Read and MacFarlane, 2006; Vanette and Krosnick, 2014). Moreover, several adult usability questionnaires have been adapted for pediatric populations through visual supports and age-appropriate wording (Brooke, 1996; Bangor et al., 2008; Sim et al., 2006; Read, 2008; Barendregt et al., 2006; Kantosalo and Riihiaho, 2019; Banker and Lauff, 2022). Importantly, many of the available instruments rely on self-report methodologies. Although self-report measures are indispensable for capturing children’s subjective experiences, their application in developmental populations requires careful methodological consideration. Children are more vulnerable than adults to response biases such as acquiescence, suggestibility, and social desirability (Bell, 2007; Soto et al., 2008). Developmental differences in cognitive, linguistic, and executive functioning may affect children’s comprehension of item wording, response scales, and abstract constructs, potentially influencing response validity (Emerson et al., 2013). These challenges may be particularly salient in younger participants and in children with borderline or lower cognitive functioning, where reduced metacognitive monitoring and executive control can increase susceptibility to systematic response patterns (Nicolaidis et al., 2020). Difficulties with understanding may lead to incorrect or incomplete responses, while the introduction of support from another person when completing self-report measures may introduce certain types of bias, such as socially desirable responding that often lead children to provide answers aligned with perceived expectations (van de Mortel, 2008), as a consequence of respondent-assistant dynamics (Finlay and Antaki, 2012; Kramer et al., 2010).

Despite the wide range of available methods to assess usability and acceptability, a significant gap remains in the available instruments: currently, there are no brief, validated and standardized tool that integrates both dimensions and are specifically designed for children and adolescents aged 4 to 18 years. Existing instruments are either too complex, requiring metacognitive and linguistic skills that are not yet fully developed, or overly simplified, resulting in reduced discriminative power and evaluative comprehensiveness (Hourcade, 2007; Markopoulos et al., 2008). This limitation is particularly pronounced in the Italian context, where, to our knowledge, no questionnaires specifically developed for the developmental age population currently encompass both dimensions.

To address this need, the present study aims to create and validate a new questionnaire, the Usability and Acceptability Scale (UAS), specifically tailored for children and adolescents aged 4 to 18. By focusing on this age group, the UAS seeks to enhance our understanding of how children engage with technology, ultimately contributing to the development of more effective and engaging digital tools. The UAS is an adapted version of the Technology Acceptance Model (TAM; Venkatesh and Bala, 2008) and System Usability Scale (SUS; Brooke, 1996) questionnaires. In this study, we administered the UAS to assess the usability and acceptability of new digital tools for neuropsychological evaluation, such as MatriKS, a computerized test measuring fluid intelligence (de Chiusole et al., 2024).

Introduced by Davis (1989), TAM is one of the most influential models for understanding user acceptance of technology. TAM has been extensively applied in various contexts, including educational technology (Dizon, 2016; Lee et al., 2009; Ratna and Mehra, 2015; Teo et al., 2008), healthcare (Nikolaus et al., 2014), and consumer software (Lilley et al., 2004), due to its easiness and predictive power (Davis et al., 1989; Lee et al., 2003). However, as technology evolves and new factors that influence user acceptance emerge, the need for more comprehensive models becomes evident. TAM2 (Venkatesh and Davis, 2000) extended the original model by including additional variables such as social influence and cognitive instrumental processes, further refining the understanding of technology adoption. Building upon this, TAM3 (Venkatesh and Bala, 2008) introduced an even more nuanced framework by integrating the determinants of perceived ease of use from the original TAM with the determinants of perceived usefulness from TAM2. TAM3 considers a wider range of factors, including computer self-efficacy, perceptions of external control, and perceived enjoyment, making it particularly suitable for evaluating complex and interactive technologies. This model provides a robust framework for assessing the likelihood of technology adoption by addressing both user perceptions and contextual factors that may influence technology acceptance (Davis et al., 1989).

The SUS (Brooke, 1996) is a widely used standardized questionnaire for measuring usability, specifically designed to generate a comprehensive single measure of perceived usability. It is known as a “quick” survey scale that allows practitioners to assess the usability of novel technologies easily and efficiently. Since its introduction, SUS has been incorporated into a variety of commercial usability toolkits and technology-based systems, including consumer software, websites, applications, and hardware products (Bangor et al., 2008; Blažica and Lewis, 2015; Brooke, 2013; Lewis, 2018; Marzuki et al., 2018).

The SUS items have been developed according to the three usability criteria defined by ISO 9241-11: (1) the ability of users to complete tasks using the system and the quality of the output of those tasks (i.e., effectiveness); (2) the level of resource consumed in performing tasks (i.e., efficiency); and (3) the users’ subjective reactions using the system (i.e., satisfaction).

The original SUS (Brooke, 1996) includes a mix of positive and negative items, and it was structured to control for acquiescence bias and to identify respondents who did not pay attention to the statements. However, several studies have highlighted the potential issues associated with the inclusion of both positive and negative items (Barnette, 2000; Stewart and Frye, 2004). For example, it can result in a reduction in internal reliability (Stewart and Frye, 2004), a distortion of the factor structure (Pilotte and Gable, 1990; Schriesheim and Hill, 1981), and an increase in the number of interpretation problems encountered when using the scale in cross-cultural contexts (Wong et al., 2003).

Moreover, mixed items may lead to difficulty in switching users’ response behaviours, and they can increase the cognitive load (Kortum et al., 2021). This could be particularly true for clinical and younger populations, whose executive functions may be impaired or still in development.

Notably, some authors suggested that questionnaires for usability assessment should avoid the inclusion of a mixture of positive and negative items and that researchers who do not specifically need to use the standard SUS should consider using the positive version to reduce the likelihood of response or scoring errors (e.g., Lewis, 2018). A version of the SUS that includes only positively worded items has been created (Sauro and Lewis, 2011) and it has been found to have satisfactory reliability, validity, and sensitivity (Lewis, 2018). Thus, the positive SUS emerges as a valuable alternative to the standard SUS, offering the advantages of reduced cognitive load and shifting ability.

Since 2014, several translations and psychometric evaluations of SUS have been published, including Arabic (AlGhannam et al., 2017), Danish (Hvidt et al., 2020), Chinese (Wang et al., 2020), French (Gronier and Baudet, 2021), Italian (Borsci et al., 2009), Malay (Marzuki et al., 2018), Persian (Dianat et al., 2014), Polish (Borkowska and Jach, 2017), Portuguese (Martins et al., 2015), Slovene (Blažica and Lewis, 2015), and other languages. In addition, translations of SUS into German (Perrig and von Felten, 2025), and Finnish (Raita and Oulasvirta, 2011) have been conducted, although there is no available data regarding their psychometric properties.

Despite its wide adoption a significant need for validations in different populations remains (Lewis, 2018).

Based on these premises, we developed and validated the UAS, a new and comprehensive Italian scale designed to assess both the usability and acceptability of a digital tool in the developmental population. Starting from assessing face validity and content validity of the scale, the present investigation aimed to validate the UAS in a group of children aged 4–18. We investigated the construct validity of the UAS, examining the factorial structure of the scale using Exploratory Factor Analysis (EFA) and Confirmatory Factor Analysis (CFA). Then we investigated the reliability of UAS measurement, checking the internal consistency of Cronbach’s *α*, McDonald’s *ω*, and corrected item-total correlation. Moreover, we verified if the factorial structure was invariant for sex and age. This study would provide evidence about the reliability and validity of this new tool for assessing the usability and acceptability of digital technologies in children and adolescents, thereby enhancing the development and implementation of child-friendly digital assessments.

## Materials and methods

### The Usability and Acceptability Scale (UAS)

**Figure 1:**
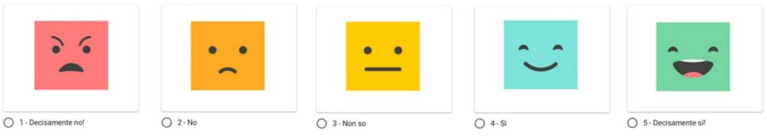
Example of verbal response option and visual response option of UAS. The verbal responses are presented in Italian. Below is the corresponding English translation: Decisamente no!, Definitely not!; No, Not; Non so., I do not know.; Sì, Agree; Decisamente sì!, Definitely yes!.

The UAS is an eight-item questionnaire, based on the Italian adaptations of the SUS (Brooke, 1996) and TAM (Davis, 1989; Venkatesh and Bala, 2008). It has been specifically designed to assess usability and acceptability in children and adolescents. It has been structured to achieve brevity and ease of comprehension, simplifying the language and using emoticons to make it easier for the younger users to understand and answer the questions. In the UAS, participants are asked to rate their agreement with eight statements using a 5-point Likert scale ranging from “Strongly Disagree” to “Strongly Agree.” Each statement is presented in a child-friendly format using simple language accompanied by icons. Specifically, the verbal response options were paired with coloured emoticon images, this visual aid was designed to increase comprehension and engagement in the rating process (see Figure 1).

In the present study, we asked 10 experts to evaluate the content validity of the questionnaire. Experts were chosen based on their expertise in the field of usability and acceptability and their experience with children in neuropsychological assessment. To ensure a structured and replicable evaluation process, each expert received a standardized package via email, including: a background questionnaire, the preliminary version of the UAS scale, an introductory letter describing the aims of the study and the target population, and a structured content validation form. Experts were asked to independently evaluate each item with respect to multiple content-related criteria, including relevance to the construct, clarity of wording, appropriateness for the target population, and comprehensibility for children. In addition to quantitative ratings, experts were invited to provide qualitative comments and to suggest item modifications, deletions, or the addition of new content areas.

All completed form were returned electronically and systematically reviewed by the research team. Expert feedback was analyzed both quantitatively (i.e., inspection of ratings across experts) and qualitatively (i.e., thematic analysis of open-ended comments). The item revision process followed a set of predefined decision rules aimed at maximizing content validity: items were retained when they were consistently judged as highly relevant and comprehensible; items were revised when experts identified issues related to wording, developmental appropriateness, or ambiguity; and items were removed when they were judged as redundant, insufficiently relevant to the target constructs, or inappropriate for children. When multiple experts suggested similar changes, these recommendations were prioritized in the revision process.

Following this expert-based review, the questionnaire underwent a structured item reduction and refinement process. The original questionnaire consisted of *N* = 22 items derived from the System Usability Scale (SUS) and the Technology Acceptance Model (TAM3) (see Supplementary Tables S1 and S2 for the original items). Based on expert evaluations, the scale was reduced to a more parsimonious version while preserving coverage of the core dimensions of usability and acceptability. This process resulted in the selection, modification, and refinement of items judged to best represent the intended constructs and to be most suitable for the developmental level of the target population. The final version of the UAS consists of eight items: four items assessing the usability dimension adapted from the SUS, and four items evaluating the acceptability dimension, specifically, the perceived ease of use as a component of acceptability, derived from TAM3 items.

In line with the recommendations of Brooke (1996), who advocated for a flexible application of the SUS items according to the specific context of interest, only four out of 10 SUS items were considered. Specifically, consistent with the positive SUS version (Sauro and Lewis, 2011; see Supplementary Table S1, for the complete version), only positive worded items were included. Namely, Items 1 (“I think that I would like to use the…frequently”), 2 (“I found the…to be simple”), 3 (“I thought the…was easy to use”), and 7 (“I would imagine that most people would learn to use the…very quickly”), as experts judged these items to be more easily comprehensible and cognitive less demanding for the targeted audience of children, thereby increasing developmental appropriateness and reducing potential response bias associated with negatively worded items. These source items were subsequently adapted and reworded for the specific context and target population, resulting in the following final usability items: 1 “I would gladly take the test again,” 2 “The test was very easy,” 3 “I think that anyone could take this test,” and 4 “It was easy to understand what to do during this test” (as shown in Table 1).

**Table 1:** Items of the final version of Usability and Acceptability Scale (UAS).

UAS scale	Italian version	English version
Item 1	Rifarei volentieri la prova	I would gladly take the test again
Item 2	La prova è stata molto facile	The test was very easy
Item 3	Penso che tutti possano fare questa prova	I think that anyone could take this test
Item 4	È stato facile capire cosa fare durante la prova	It was easy to understand what to do during this test
Item 5	Penso che fare la prova sul tablet sia stato semplice	I think that taking the test on the tablet was easy
Item 6	Credo che per tutti sia facile fare la prova con il tablet	I think that it would be easy for anyone to take the test on the tablet
Item 7	Sono stato bene a fare le prove sul tablet	I felt good taking the test on the tablet
Item 8	Vorrei usare sempre il tablet per fare questa prova	I would like to always use the tablet for this test

A similar theoretically and developmentally informed approach was applied to the selection of acceptability items. The original TAM version consists of 12 items, with six evaluating perceived usefulness (PU) and six assessing 9 perceived ease of use (PEU; see Supplementary Table S2, for the complete list of original items). PU refers to the degree to which a person believes that technology will enhance job performance, while PEU is defined as the extent to which a person believes that using technology will be effortless (Davis, 1989). Given that the UAS was designed to assess the usability and acceptability of MatriKS, a new digital assessment tool for assessing fluid intelligence intended to support the job performance of clinicians and experimenters, rather than to improve the performance of the tested participants. PU items were judged by experts as conceptually inappropriate for the test-taker population. Accordingly, only PEU related items were considered relevant for inclusion.

Within the PEU items, a further expert-guided selection was performed to retain items that best captured children’s direct experience with the tool while minimizing redundancy and cognitive load. Four PEU items were ultimately selected: the UAS questionnaire selectively incorporated just four items. Specifically, Specifically, Items 5 (“Using…would make it easier to do my job”) and 6 (“I would find…useful in my job”) pertained to the perceived ease of use for oneself and others, Item 9 (“My interaction with the…is clear and understandable”) and Item 7 (“I find the…to be easy to use”) captured core aspects of perceived ease of use, Item 11 (“I find using the…to be enjoyable”) focused on perceived pleasantness/fun, and Item 12 (“Assuming I had access to the…, I intend to use it”) addressed intentionality. These source items were subsequently adapted and reworded for the specific tablet-based testing context and for child respondents, resulting in the following final acceptability items: 5 “I think that taking the test on the tablet was easy,” 6 “I think that it would be easy for anyone to take the test on the tablet,” 7 “I felt good taking the test on the tablet,” and 8 “I would like to always use the tablet for this test.” The full mapping between source and final UAS items is reported in the Supplementary Tables S1 and S2. Additionally, to the expert-based content validation, face validity was further examined through direct input from the target population. Face validity was defined as the extent to which the scale’s purpose is apparent to the target users and appears to be an appropriate measure for its intended purpose (Salkind, 2010; Mosier, 1947), encompassing aspects such as feasibility, readability, consistency in style and formatting, and the clarity of the language used (DeVon et al., 2007). A sample of *N* = 10 participants from the target population was asked to select the statement they thought best reflected the construct in question and to change the wording, delete statements, or add new aspects they thought relevant. Participants were also invited to suggest wording changes, delete unclear statements, and propose additional aspects they considered relevant. Feedback from this phase was used to further refine item wording and to ensure that the final version of the UAS was not only theoretically sound but also developmentally appropriate and easily interpretable by children. Overall, the iterative combination of expert review and target-user feedback resulted in a concise, developmentally sensitive instrument with enhanced evidence of content and face validity, while maintaining conceptual coverage of the usability and acceptability constructs relevant to the evaluation of MatriKS. The adaptation process, including linguistic simplification and item reduction, was finalized prior to the factorial analyses. The EFA and CFA were conducted on a split sample using the same predefined version of the scale, and no post-hoc modifications were made following the EFA.

The final adapted version of the scale (UAS) is publicly available and is freely accessible for research purposes.

### Procedure

In this study, we administered the UAS to assess the usability and acceptability of MatriKS, a new digital tool for the assessment of fluid intelligence based on the theory of knowledge structures (Doignon and Falmagne, 1985; Falmagne and Doignon, 2010; Heller and Stefanutti, 2024). MatriKS is available in two versions, differentiated according to participants’ age, namely between 4 and 11 years old and 12 or more years old. Participants completed a different version of MatriKS according to their chronological age. The test is part of PsycAssist,^1^ a platform for the assessment of neuropsychological functioning (de Chiusole et al., 2024).

Participants completed first MatriKS and then the UAS using a tablet (IoS operating system with a 10.9-inch screen). All participants completed the questionnaire independently, except for the youngest children, aged 4 to 6. For these children, examiners assisted by reading the items aloud, as the children’s reading skills were not yet fully developed. Children then responded autonomously to the items, with visual support provided by emoticons.

### Participants

The UAS was administered to a total sample of *N* = 908 participants of the general population aged 4 to 18 (Female = 53%, Male = 47%, Mean age: 10.53 ± 3.50). The descriptive statistics of the general sample are reported in Table 2. Participants were randomly divided into two groups to conduct the EFA (EFA group, *n* = 359) and CFA (CFA group, *n* = 549). The two groups did not differ in terms of age (*t*_(906)_ = −0.29; *p* = 0.79), sex (*χ*^2^_(1)_ = 1.30; *p* = 0.25), or educational level (*χ*^2^_(3)_ = 0.63; *p* = 0.89). The sample size of the two groups was established *a priori* according to the minimum criteria to have a subject to an item ratio of 10:1 in the EFA (Nunnally, 1978) and at least 10 observations for each freely estimated model parameter in the CFA (Kline, 1998, 2023). The data were collected in different regions of the North, Centre, and South of Italy. Schools of all levels were involved, from kindergarten to high school. The school principals were contacted in advance to inform them about the aim of the project and to inquire about their willingness to participate in the project. Subsequently, the parents or legal guardians of the children enrolled in the participating classrooms were provided with the informed consent in accordance with the Declaration of Helsinki recommendations (Williams, 2008). Only children whose parents provided informed consent were included in the study, and subsequently, the children themselves decided whether or not to participate. The exclusion criteria were the presence of motor, visual, and auditory impairments that prevented the participants from completing the task and the questionnaire. An expert team of psychologists handled the assessments and managed communications with both teachers and parents.

**Table 2:** Descriptive statistics of the total sample of participants (*N* = 908) and Exploratory Factor Analysis (EFA) and Confirmatory Factor Analysis (CFA) groups.

Variables	Total sample (*n* = 908)	EFA group (*n* = 359)	CFA group (*n* = 549)
Sex (*n*)			
M-F	47–53	49–51	46–54
Age (years)			
Range	4–18	4–18	4–18
*M* (SD)	10.52 (3.50)	10.01 (3.45)	10.91 (3.48)
Educational level (*n*)			
Kindergarten	107	42	65
Primary school	346	142	204
Middle School	251	98	153
High School	204	77	127

M-F, male–female; *M* (SD), mean (standard deviation).

The study was approved by the ethical committee for the psychological research of the University of Padua (protocol code E047A9B0520E9732A8365DC72335EE90/ date of approval: 8 July, 20222).

## Data analysis

All statistical analyses were performed on the variables related to the final version of the UAS (see Table 1 for details on items) using IBM SPSS 25 (SPSS Inc., Chicago, IL, United States) and JASP version 0.18.3.0 (JASP Team, 2024).

As suggested by Tabachnick and Fidell (2013), for the EFA and CFA groups we checked for the presence of univariate outliers (scores more than 3.29 standard deviations above or below the corresponding group mean were excluded), normalized the UAS total score distribution, and checked for the presence of multivariate outliers. Each time we excluded participants, we normalized the UAS score distribution.

### Exploratory Factor Analysis

The Exploratory Factor Analysis (EFA) was conducted using the Weighted Least Squares (WLS) technique, which aims to capture most of the variance across a set of variables with a reduced number of factors (Fabrigar and Wegener, 2012). An oblimin rotation was applied, suitable when factors are expected to be correlated (Fabrigar et al., 1999; Costello and Osborne, 2005).

Parallel analysis, scree test, incremental variance, and interpretability of the pattern of factor loadings were employed to choose the number of factors to retain (Fabrigar et al., 1999). Items with loadings greater than 0.40 and cross-loadings less than 0.10 were considered for inclusion in a factor.

### Confirmatory Factor Analysis

The goodness of fit of the factorial structure of the scale identified in the EFA was tested. Maximum likelihood estimation with a mean-adjusted Chi-square test (MLM estimator), which is robust to non-normal score distributions, was used. The metric of the latent variables was set by fixing the factor loading of the first item to one, for each factor. Overall model fit was determined by using the Satorra-Bentler scaled Chi-square statistic (S-B*χ*^2^), robust comparative fit index (rCFI), robust root mean square error of approximation (rRMSEA) with associated 95% confidence intervals (CIs), and standardized root mean square residual (SRMR) (Schermelleh-Engel et al., 2003). Values close to 0.95 for rCFI, smaller than 0.05 for rRMSEA, and smaller than 0.08 for SRMR suggest a reasonable fit (Byrne, 2013).

The factorial structure found in EFA was compared with alternative nested models, which were theoretically plausible. To this aim, ΔS-B*χ*^2^ and ΔrCFI were used as fit indices. To indicate that the null hypothesis of equivalence should be rejected (i.e., the EFA factorial structure model had a better fit than the alternative model), a significant ΔS-B*χ*^2^ and a value of ΔrCFI (which is less affected by sample size) higher than 0.01 are required (Cheung and Rensvold, 2002). Finally, Akaike’s information criterion (AIC) was calculated, with a lower value indicating a better fit of the model to the data (Schermelleh-Engel et al., 2003; Sterba and Pek, 2012).

### Measurement invariance

To verify the measurement invariance of the UAS, we tested the model across the sex of participants (female vs. male) and across three age groups (4–6 years, 7–11 years and 12–18 years), performing three levels of invariance: configural, metric, and scalar (i.e., same item intercepts) (Vandenberg and Lance, 2000). Measurement invariance was assessed specifically within the CFA subsample.

For the assessment of configural invariance, no constraints were imposed, enabling a test of whether the pattern of fixed and freely estimated parameters remained consistent across sub-groups. Regarding the metric invariance (weak invariance), the factor loadings of each item on the corresponding factor (i.e., the scale unit and the metric of each item) were constrained to be invariant across subgroups. Moreover, we performed the scalar invariance (strong invariance) where the factor loadings plus the intercepts of each item on the corresponding factor (i.e., the origin of the scale of each item) were constrained to be invariant (Byrne, 2008; Vandenberg and Lance, 2000).

### Sensitivity analyses

To examine potential effects of administration heterogeneity, we conducted sensitivity analyses comparing examiner-assisted administration (children aged 4–6 years) with non-assisted administration. Full details of these analyses are provided in the Supplementary materials.

### Internal consistency

As a measure of internal consistency Cronbach’s *α* (cutoff ≥0.70; Nunnally, 1978), McDonald’s *ω* (cutoff ≥0.70; McDonald, 1999), and corrected item-total correlations (cutoff ≥ 0.30; Norman and Streiner, 2008) were computed for each factor and for the total scale (Dunn et al., 2014; Soĉan, 2000). These measures were assessed within the total sample.

## Results

### Exploratory Factor Analysis (EFA)

The EFA was conducted on the first subsample (*n* = 359). Using parallel analysis, scree test, incremental variance, and interpretability of item factor loadings, all methods consistently extracted two interrelated factors, which were then rotated using oblimin rotation. The total explained variance was 42%. All items met the inclusion criteria for factor retention, with loadings greater than 0.40 and cross-loadings less than 0.10. Table 3 presents the item loadings for the two-factor solution (Model 1). The first factor consists of four items related to usability (items 1, 2, 3, and 4), while the second factor includes four items representing acceptability (items 5, 6, 7, and 8). Skewness and kurtosis computed on the two-factor scores indicated approximately normal univariate distributions, as all values were lower than |2| (Tabachnick and Fidell, 2013); skewness ranged from −1.347 to 0.149 (SE = 0.18), and kurtosis ranged from −0.853 to 3.153 (SE = 0.357).

**Table 3:** Exploratory Factor Analysis (EFA) results.

EFA factor loadings (*n* = 359)			
Item content	Mean ± *SD*	F1	F2
1. I would gladly take the test again	3.95 ± 0.88	**0.694**	0.010
2. The test was very easy	3.59 ± 0.98	**0.771**	−0.031
3. I think that anyone could take this test	3.71 ± 0.97	**0.456**	0.002
4. It was easy to understand what to do during this test	3.94 ± 0.96	**0.689**	0.055
5. I think that taking the test on the tablet was easy	4.34 ± 0.67	0.100	**0.584**
6. I think that it would be easy for anyone to take the test on the tablet	3.81 ± 0.98	−0.047	**0.548**
7. I felt good taking the test on the tablet	4.46 ± 0.61	0.082	**0.641**
8. I would like to always use the tablet for this test	4.15 ± 0.87	−0.091	**0.661**
**Eigenvalues after oblimin rotation**		1.8 (23%)	1.1(19%)

Bold values represent the factor loadings associated with the two factors.

### Confirmatory Factor Analysis (CFA)

**Figure 2:**
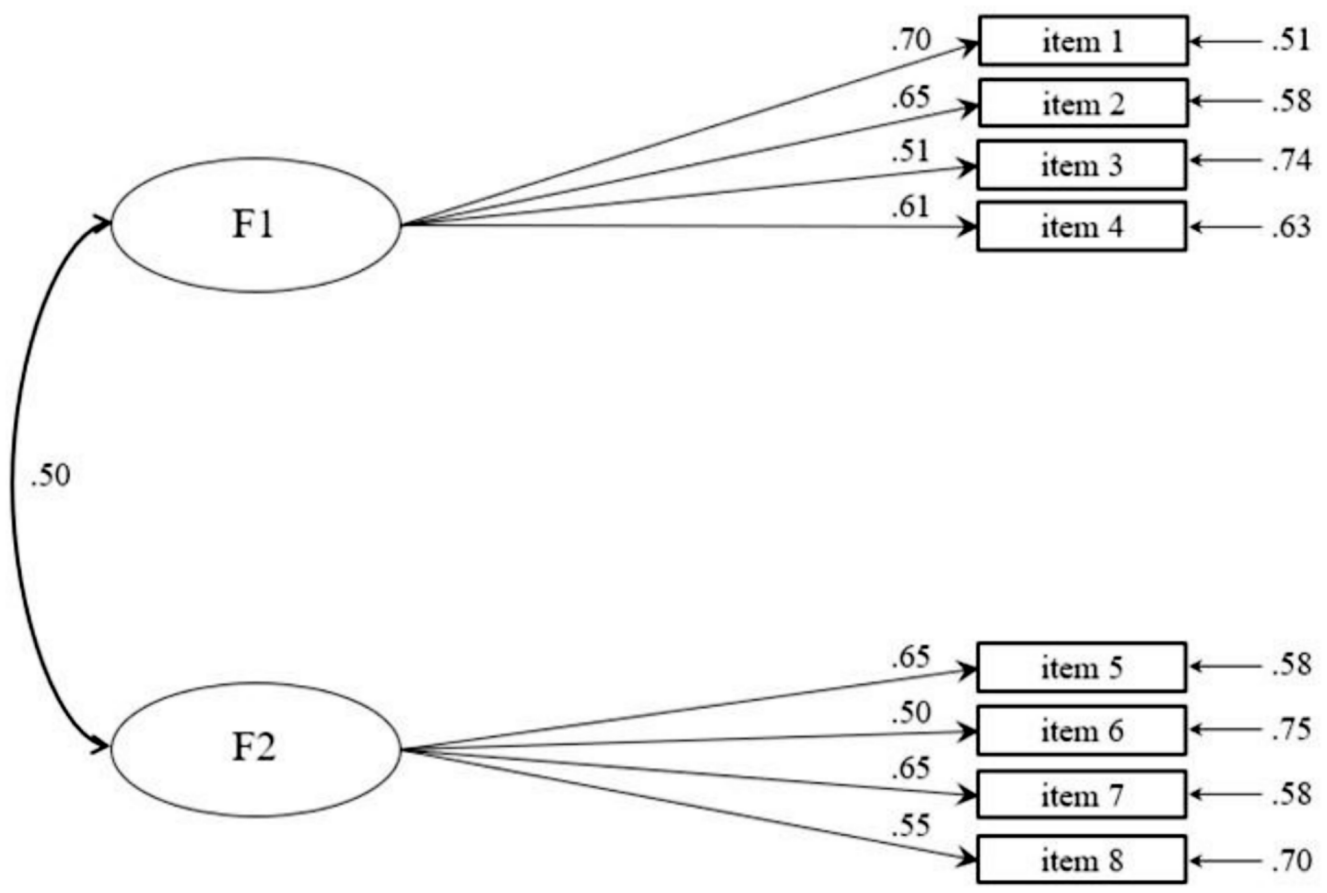
Measurement model with standardized parameters (*n* = 549).

The two-factor model selected in the EFA was tested on the second subsample (*n* = 549) using CFA. Results indicated marginal to acceptable fit to the data, with all indices close to the expected value: S-B*χ*^2^ *=* 89,76, *p* < 0.05; rCFI = 0.92; rRMSEA = 0.082, 95% CI [0.066, 0.100]; SRMR = 0.044. It should be noted, however, that the rCFI value does not reach the conventional threshold of 0.95 and the rRMSEA slightly exceeds the recommended cutoff of 0.08, indicating a marginal—yet still acceptable—model fit. Overall, the pattern of indices suggests an adequate representation of the data. Aiming to evaluate possible alternative models explaining specificity or overlapping between the investigated domains, the two-factor model obtained by EFA (Model 1, where the items of the questionnaire cluster around domains of usability and acceptability) was compared to a one-factor model solution (Model 2, representing unique general factor in which all items of the UAS were loaded on a single dimension). The CFA findings strongly support the validity of the UAS’s two-factor structure showing that Model 1 was the most representative model of the questionnaire structure with the best fit and better results than the alternative nested model: ΔS-B*χ*^2^ (Δd*f*) range = 253.718 (20)–89.76 (19); ΔrCFI range = 0.72–0.91. Concerning the other parsimony fit indices, it is possible to note as the two-factor model (Model 1) showed the lowest values for AIC (Model 1 = 10424.668; Model 2 = 10586.617) confirming that Model 1 turned out to be the model that best fits the data and best explains the dimensionality of the analyzed data. All factor loadings were statistically significant. Each item loaded highly (>0.50) and significantly (*p* < 0.001) on its designated factor, with factor loadings from 0.50 to 0.70 (see Figure 2). Skewness and kurtosis computed on the CFA subsample indicated approximately normal univariate distributions, as all values were lower than |2| (Tabachnick and Fidell, 2013); skewness ranged from −1.153 to −0.338 (SE = 0.104), and kurtosis ranged from −0.735 to 1.128 (*SE* = 0.208).

### Measurement invariance across sex

The two-factor model exhibited full configural, metric, and scalar invariance (Table 4 presents the results of the sex invariance test, and fit indices for the model across males and females). All factor loadings were statistically significant and comparable between groups (*p* < 0.001). The models assessing configural, metric, and scalar invariance demonstrated good fit indices that did not vary significantly from one model to the others. The UAS showed configural, metric, and scalar invariance across sexes (see Table 4), indicating that latent structure, factor loadings, and intercepts are equivalent for both groups.

**Table 4:** Results of the measurement invariance test across groups (sex: male vs. female).

Model	GFI	NFI	PNFI	SRMR	RMSEA
Model (a)—configural: factor structure constrained to be equal	0.998	0.881	0.598	0.050	0.082
Model (b)—metric: factor loadings constrained to be equal	0.998	0876	0.735	0.052	0.072
Model (c)—scalar: item intercepts constrained to be equal	0.997	0.860	0.844	0.050	0.069

GFI, Goodness of Fit Index; NFI, Normed Fit Index; PNFI, Parsimony-Adjusted Normed Fit Index; SRMR, standardized root means square residual; RMSEA, root mean square error of approximation.

### Measurement invariance across age

Three nested models with progressively stringent constraints were evaluated to test the invariance across three age groups 4–6 years (preschool/early childhood), 7–11 years (middle childhood), and 12–18 years (adolescence): configural invariance (Model 1), metric invariance (Model 2), and scalar invariance (Model 3). All factor loadings were statistically significant within each group and showed comparable magnitude across age groups. Table 5 presents the results of the age invariance analyses and model fit indices.

**Table 5:** Results of the measurement invariance test across age groups (4–6, 7–11, 12+ years).

Model	GFI	NFI	PNFI	SRMR	RMSEA
Model (a)—configural: factor structure constrained to be equal	0.997	0.830	0.563	0.055	0.091
Model (b)—metric: factor loadings constrained to be equal	0.997	0804	0.689	0.067	0.084
Model (c)—scalar: item intercepts constrained to be equal	0.991	0.804	0.479	0.220	0.152

GFI, Goodness of Fit Index; NFI, Normed Fit Index; PNFI, Parsimony-Adjusted Normed Fit Index; SRMR, Standardized root means square residual; RMSEA, root mean square error of approximation.

Results demonstrated acceptable configural invariance (GFI = 0.997, NFI = 0.830, SRMR = 0.055, RMSEA = 0.091), indicating that the basic two-factor structure is consistent across developmental stages. Metric invariance was also supported, as constraining factor loadings to equality did not significantly worsen model fit (Δ*χ*^2^(15) = 21.95, *p* = 0.109). This finding suggests that the relationship between observed indicators and latent constructs is stable across age groups and that the questionnaire items retain comparable meaning across developmental periods. In contrast, scalar invariance was not supported, as constraining item intercepts to equality resulted in a significant decrease in model fit (Δ*χ*^2^(16) = 292.39, *p* < 0.001). Fit indices for the scalar model (GFI = 0.991, NFI = 0.804, PNFI = 0.479, SRMR = 0.220, RMSEA = 0.152) further indicated reduced model fit, suggesting that item intercepts may differ across age groups.

### Internal consistency

The internal consistency of the UAS was assessed using both Cronbach’s *α* and McDonald’s *ω*, yielding promising results. The overall reliability was found to be acceptable, with Cronbach’s *α* at 0.74 and McDonald’s *ω* at 0.77, suggesting that the scale is internally consistent. When examining the individual factors, Factor 1 (F1) showed solid reliability with McDonald’s *ω* at 0.71 and Cronbach’s *α* at 0.70. However, Factor 2 (F2) had slightly lower internal consistency, with McDonald’s *ω* at 0.65 and Cronbach’s *α* at 0.66. While these values indicate somewhat lower reliability, they still fall within acceptable ranges, suggesting that both factors can be considered reliable, though further investigation might be warranted for Factor 2.

### Sensitivity analyses

Sensitivity analyses examining potential effects of administration heterogeneity (examiner-assisted for children aged 4–6 years vs. non-assisted administration) showed no significant differences in the total usability score. Small but statistically significant differences emerged at the factor level, in opposite directions across F1 and F2. Reliability indices were comparable across modalities, indicating stable internal consistency. Complete statistical results and a more extensive discussion of these findings are provided in the Supplementary materials.

## Discussion and conclusion

This study aimed to validate and analyze the psychometric properties of the Usability and Attitude Scale (UAS), a questionnaire designed to assess the usability and acceptability of digital technologies among children and adolescents.

Our findings from EFA and CFA suggest that the UAS is a reliable and valid tool for evaluating usability and acceptability among children aged 4 to 18 years. The EFA revealed a clear factor structure for the UAS, indicating distinct dimensions for usability and acceptability. The results showed that the items loaded appropriately onto the hypothesized factors, with eigenvalues supporting a two-factor solution. This is consistent with the theoretical foundations of the Technology Acceptance Model (TAM) and System Usability Scale (SUS), which posit that usability and attitude toward technology are critical determinants of user acceptance. The CFA confirmed the two-factor model structure identified in the EFA, with fit indices indicating a good model fit. These findings reinforce the scale’s structural validity and suggest that the UAS is a reliable instrument for measuring the constructs it was designed to assess. The internal consistency, as indicated by Cronbach’s *α*, was overall satisfactory, providing further evidence of the reliability of the scale. However, with respect to the second factor, reliability indices were slightly below the conventional threshold and should therefore be interpreted with some caution. This may be partly attributable to the reduced number of items comprising this subscale, given that internal consistency coefficients such as Cronbach’s *α* are sensitive to scale length. Nevertheless, this does not substantially undermine the overall reliability of the scale, as the values remain close to recommended standards and the factor structure is theoretically coherent. With respect to the sensitivity analyses conducted to evaluate administration heterogeneity, findings indicate that administration modality does not meaningfully affect the overall usability score. Although small differences were observed at the factor level, these effects were limited in magnitude and did not impact internal consistency. Overall, the results support the robustness of the instrument’s psychometric properties across administration formats, suggesting that the principal validation findings remain stable regardless of examiner assistance.

Our study aligns with previous research highlighting the importance of usability and acceptability in the adoption of digital technologies by children. The TAM framework has been extensively validated in adult populations, but its application to children is relatively novel. The successful adaptation and validation of the UAS demonstrate that these theoretical constructs are applicable and relevant to younger users as well.

The development and validation of the UAS for children represents a significant advancement in the assessment of digital technologies tailored for younger users. The UAS could be a valuable tool for developers, educators, and researchers engaged in the implementation of digital technologies for children. It provides a reliable measure of usability and acceptability, thus enabling the identification of strengths and improvement areas in digital products with the goal of better meeting the needs of young users. This can enhance the effectiveness of educational and therapeutic interventions delivered through digital platforms. For developers, the UAS provides insights that guide the design of user-friendly and engaging products. For educators and researchers, it assesses the impact of digital technologies on learning outcomes and engagement, promoting the evidence-based integration of technology into educational settings. Beyond structural validity, the present study examined the invariance across sex and age groups, a critical property for a scale intended for use across diverse developmental populations. Regarding sex invariance, the two-factor model showed full configural, metric, and scalar invariance across males and females, indicating that the latent structure, factor loadings, and item intercepts are equivalent between groups. These findings suggest that the UAS captures the same constructs in the same way regardless of sex, and that meaningful comparisons of latent mean scores between males and females are statistically justified. Regarding age invariance, results revealed a pattern of partial measurement invariance across the three developmental groups (4–6, 7–11, and 12–18 years). Configural invariance was supported, confirming that the two-factor structure of the UAS is consistent across developmental stages. Metric invariance was also established, as constraining factor loadings to equality across groups did not significantly worsen model fit, indicating that the relationship between observed items and their underlying latent constructs is stable across developmental periods and that the questionnaire items retain comparable meaning from early childhood through adolescence. However, scalar invariance was not supported, as the equality constraints placed on item intercepts resulted in a significant deterioration of model fit. This finding suggests that children at different developmental stages may systematically endorse items at different levels, even when their standing on the underlying latent construct is equivalent. Such differences in item intercepts are theoretically plausible given the broad developmental span covered by the UAS and are consistent with changes in cognitive maturity, linguistic comprehension, and response tendencies across childhood and adolescence. Importantly, because metric invariance was achieved, the latent constructs retain the same substantive meaning across age groups, and the factorial structure of the scale remains stable. Caution is nonetheless warranted when making direct mean-level comparisons across developmental stages. Taken together, the invariance analyses provide robust evidence for the cross-group comparability of the UAS across sex and support its structural equivalence across developmental stages, strengthening its utility as a standardized assessment tool for children and adolescents.

Although the UAS demonstrates strong psychometric properties, we acknowledge some study limitations that warrant consideration. The data collection, though in diverse regions, was limited to Italy, and cultural factors may influence the generalizability of the findings. Therefore, future research should explore the applicability of the UAS in different cultural contexts to ensure its broader validity. A further limitation concerns the specific digital technology used for validation. The UAS has been tested exclusively with MatriKS, and additional studies are needed to verify its applicability and psychometric properties with other digital tools. Testing the scale across diverse digital platforms and applications will be essential to establish its generalizability and confirm its utility as a reliable measure of usability and acceptability for children’s digital technologies.

Moreover, convergent and criterion validity were not assessed in this study. Currently, in the Italian context, no brief and easily administrable instruments exist for developmental populations that simultaneously capture both usability and acceptability of digital tools, which limits the possibility of formally evaluating convergent validity5. Nevertheless, establishing convergent validity is a crucial step to further confirm the construct validity of the UAS. Future research should aim to compare UAS scores with validated measures of related constructs, such as the System Usability Scale (SUS) or Technology Acceptance Model (TAM) based instruments, once appropriate child-adapted Italian versions become available. Conducting such analyses would allow us to determine whether UAS scores converge with theoretically aligned constructs, providing robust empirical support for its validity in assessing children’s interactions with digital technologies. Additionally, the current validation was conducted exclusively in educational settings, aware that to enhance the ecological validity and broaden the applicability of the UAS beyond educational settings, it would be valuable to validate the instrument in different contexts, such as clinical and therapeutic environments. We are planning to test the UAS with clinical populations, which will provide important insights into its applicability for assessing digital technologies used in assessment and intervention contexts.

Additionally, while the UAS captures key aspects of usability and acceptability, it may benefit from further refinement and expansion to include additional dimensions such as long-term engagement and user satisfaction. Moreover, longitudinal studies could provide important insights into how these perceptions evolve over time and with prolonged use of digital technologies. Despite these limitations, the UAS offers a novel and practical tool for evaluating usability and acceptability in children, addressing a gap in the current assessment landscape.

To conclude, our results support the two-factor structure of the UAS, aligning with the theoretical constructs of usability and acceptability. The EFA results support a two-factor structure for the UAS, capturing distinct but related constructs of usability and acceptability. The identified factors are both meaningful and interpretable, reflecting the theoretical underpinnings of the scale. The CFA results provide robust evidence supporting the validity and reliability of the two-factor structure of the UAS, making it a valuable instrument for evaluating digital tools designed for children. The good internal consistency and significant factor loadings indicate that the UAS could be a reliable tool for assessing children’s usability and attitudes toward digital tools like MatriKS.

The development and validation of the UAS represent a significant contribution to the field of child-centred digital technology assessment. By providing a reliable and valid measure of usability and acceptability, the UAS helps ensure that digital technologies designed for children are both effective and engaging. This, in turn, can enhance the educational and developmental outcomes associated with the use of these technologies, ultimately contributing to better learning experiences and improved quality of life for young users.

## Data Availability

The raw data supporting the conclusions of this article will be made available by the authors, without undue reservation.

## Footnotes

1
https://psycassist.fisppa.unipd.it/en/
