# Supplementary material for: Psychometric properties of the Usability and Acceptability Scale (UAS) for evaluating digital tools in children and adolescent users
Source: Front Psychol. 2026 Apr 7;17:1702085. doi: 10.3389/fpsyg.2026.1702085 (PMC13096095; doi:10.3389/fpsyg.2026.1702085)
Supplement: Supplementary file 1 [file Table_1.docx]

**Supplementary Materials**

**Additional analyses and tables supporting the main manuscript.**

**Sensitivity analyses by administration modality (assisted vs non-assisted groups)**

To examine potential effects of administration heterogeneity, sensitivity analyses were performed comparing children aged 4–6 who received examiner-assisted administration versus older children with non-assisted administration. Analyses focused on the total usability score and the two factor-level scores (Factor 1 [F1] and Factor 2 [F2]). Independent-samples t-tests were conducted for each outcome. Levene’s test was used to assess homogeneity of variances. Effect sizes were calculated using Cohen’s d, with 95% confidence intervals. Internal consistency (Cronbach’s α and McDonald’s ω) was also computed separately for each administration modality to evaluate potential impacts on reliability.

For the total score, no significant difference emerged for the total usability score, t(906) = 0.23, p = .815; Levene’s test for homogeneity of variances was non-significant (F = 0.018, p = .894). The effect size was negligible (Cohen’s d = .02; 95% CI [−.16, .20]). However, evaluating the subscales scores, small but statistically significant differences were observed: for Factor 1 (F1), the assisted group scored higher (t(906) = 3.24, p = .001), with Cohen’s d = .30 (95% CI [.12, .48]). For Factor 2 (F2), the non-assisted group scored higher (t(906) = −3.71, p < .001), with Cohen’s d = −.34 (95% CI [−.52, −.16]). Importantly, these effects are small–moderate in magnitude and occur in opposite directions, producing a near-null effect on the aggregated total score.

Relating the internal consistency, reliability estimates support stability of the factor structure: overall Cronbach’s α = .74 and McDonald’s ω = .77; for the factors level, F1: α = .70, ω = .71; F2: α = .66, ω = .65. When considered by administration modality, Cronbach’s α for F1 was .66 (assisted) vs .72 (non-assisted), and for F2 was .65 (assisted) vs .64 (non-assisted). Minimal differences that do not indicate a substantial alteration in internal consistency. Taken together, results indicate limited domain-specific administration effects: while small and opposite differences appear at the subscale level, they do not meaningfully affect the overall usability score nor the internal consistency of the instrument. Therefore, the psychometric properties and the principal conclusions of the validation remain robust with respect to administration modality.

**Tables**

**Table S1.** Items of the Original Version of System Usability Scale (SUS; Brooke, 1996) and Items of the Positive version of System Usability Scale (Sauro & Lewis, 2011)

| Usability Scale | Original Version | Positive Version |
| --- | --- | --- |
| Item 1 | I think that I would like to use the system frequently. | I think that I would like to use the website frequently. |
| Item 2 | I found the system unnecessarily complex. | I found the website to be simple. |
| Item 3 | I thought the system was easy to use. | I thought the website was easy to use. |
| Item 4 | I think that I would need the support of a technical person to be able to use this system. | I think that I could use the website without the support of a technical person. |
| Item 5 | I found the various functions in this system were well integrated. | I found the various functions in the website were well integrated. |
| Item 6 | I thought there was too much inconsistency in this system. | I thought there was a lot of consistency in the website. |
| Item 7 | I would imagine that most people would learn to use this system very quickly. | I would imagine that most people would learn to use the website very quickly. |
| Item 8 | I found the system very cumbersome to use. | I found the website very intuitive. |
| Item 9 | I felt very confident using the system. | I felt very confident using the website. |
| Item 10 | I needed to learn a lot of things before I could get going with this system. | I could use the website without having to learn anything new. |

The response options, arranged from the left to right, are Strongly Disagree (1) to Strongly Agree (5).

**Table S2.** Items of the Original Version of Technology Acceptance Model Scale (TAM3; Venkatesh & Bala, 2008) measuring Perceived Usefulness (PU) and Perceived Ease of Use (PEU)

| TAM3 | Original Version |
| --- | --- |
| Item 1 (PU) | Using … in my job would enable me to accomplish tasks more quickly. |
| Item 2 (PU) | Using … would improve my job performance. |
| Item 3 (PU) | Using … in my job would increase my productivity |
| Item 4 (PU) | Using ... would enhance my effectiveness on the job. |
| Item 5 (PU) | Using ... would make it easier to do my job. |
| Item 6 (PU) | I would find ... useful in my job. |
| Item 7 (PEU) | I find the … to be easy to use. |
| Item 8 (PEU) | Interacting with the … does not require a lot of my mental effort |
| Item 9 (PEU) | My interaction with the … is clear and understandable. |
| Item 10 (PEU) | I would find ... to be flexible to interact with. |
| Item 11 (PEU) | I find using the … to be enjoyable. |
| Item 12 (PEU) | Assuming I had access to the …, I intend to use it. |
